# Dynamics of the Bell Prover, II

**DOI:** 10.6028/jres.095.004

**Published:** 1990

**Authors:** Fillmer W. Ruegg, Fillmer C. Ruegg

**Affiliations:** National Institute of Standards and Technology, Gaithersburg, MD 20899

**Keywords:** bell prover design, calibration procedures, flow meter calibration, flow rate, gas flow measurement, gas flow standard, prover

## Abstract

The bell prover is widely used for gas flowrate measurements by timing a known stroke of the bell as it rises, presumably with a constant speed, from a bath of sealing liquid. A differential equation for the bell motion (from the previous paper of the same title) is used together with the previous equations of motion for the gas and sealing liquid modified here to provide the basis for a computer simulation of the prover performance. Examples of the results of the computations show substantial fluctuations in all of the motions and modified measurement procedures for improved accuracy are discussed. Proposed modifications to the prover itself are shown by the computations to dampen the fluctuations and to improve the measurement accuracy. Only a limited number of changes of the prover design and of the initial conditions were researched for their effect.

## 1. Introduction

**Figure 1:**
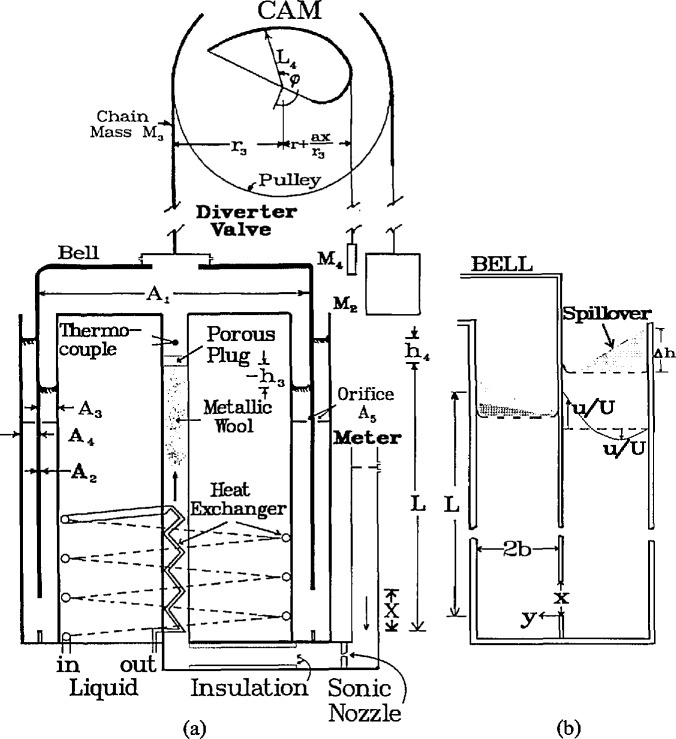
**(a)** Schematic drawing of the bell prover. **(b)** Details of liquid seal and its velocity profile.

The bell prover of figure 1(a) is essentially an ordinary prover which has been modified with parts added in an attempt to provide the basis for an improvement in gasflow measurement accuracy. A porous plug, a heat exchanger, loose metallic-wool (throughout the pipe volume *V*_b_), and thermal insulation are installed in the inlet pipe. These are suggested to help attenuate the fluctuations of pressure that are generated in the bell and also to assure equality of temperature of the bell and of the gas input. A second essential addition consists of two annular orifice plates mounted in the two sealing liquid spaces between the bell and its tank. It’s expected that the constricted liquid flow area there will help to attenuate the liquid motion which is shown in the subsequent computations herein to be a major source of flow measurement inaccuracy.

Three other additions of lesser importance are shown in figure 1(a) and these are suggested for use with both the modified and ordinary prover systems. The first is the utilization of a sonic flow nozzle to be placed somewhere in the bottom of the inlet pipe. It will serve to prevent fluctuations in the gas motion that are generated in the bell from feeding back to the meter thereby causing uncertainties in its calibration. A second benefit is that it eliminates the need to include, perhaps, a different pipe volume with the provers tare volume *V_i_* each time a different meter is installed for calibration. A third benefit arises in that the computations that are to follow here will be more likely to represent actual events for this restricted system as compared to the usual large complicated piping system.

More than one nozzle (size) may be needed to be able to cover the full range of flow of any one prover. Other basic requirements are that, at the flow rate of choice, the gas velocity in the nozzle throat be equal to the velocity of sound there, and that the gas at the entry point to bell be at the temperature of the bell. Another concept may prove useful here. Simultaneous measurements of temperature and pressure can be made at the sonic nozzles to calibrate them along with the meter. These calibrated nozzles then should prove useful as secondary flow standards for calibration of meters without the necessity of always exercising the bell prover. A second addition to the ordinary prover should include a thermocouple placed at the exit of the inlet pipe so that it can provide a confirmation that the gas and bell are at equal temperatures all during the measurement stroke of the bell. An average of measurements taken at different times and even places in the bell may not be sufficiently accurate if bell and gas temperatures are not equal.

The third difference between the modified and ordinary prover is in the location and design of the diverter valve. It is placed on top of the bell instead of on that portion of the inlet pipe that is external to the prover’s tank. It is proposed that it be an electro-mechanical device designed to be able to remotely select and replicate the stroke time and flow rate history of the gas through the valve. A lateral discharge of the gas from four ports spaced 90° around the new valve will be necessary to avoid a variable and disturbing force on the bell during its upward motion. A rotatable collar or other device with equally spaced and appropriately sized ports together with, possibly, a spring and/or an electric and/or a pneumatic motor to rotate the collar, will be needed to complete the essential parts of the valve. It’s not necessary that the new valve be located as shown in figure 1(a), but at that location the initial balance and start-up procedures do not need to differ from that ordinarily used. If it is put on the external pipe of the modified prover some small changes in the initial balance procedure and in the gas flow equations would be needed.

The differential equation of motion for the bell from reference [1], together with modified equations of motion for the gas and sealing liquid, will be used to provide the basis to derive and compare the performance of the two provers.

Special integrals of the Navier-Stokes (NS) equation will be used (for both steady and unsteady motion of the bell in the sealing liquid) to derive the liquid drag forces for use in the bell’s equation of motion. Forces connected with entrained liquid and effects not included in the special integrals will be estimated for the same application. The NS equation also provides a justification to treat the motion of the sealing liquid as if it is a “solid” and thus a one-dimensional, second-order differential equation is used for the liquid motion to calculate its effect on the gas volume under the bell.

Each set of differential equations will be subjected to numerical integration using the Runge-Kutta fourth-order method outlined in [2].

## 2. Equations of Motion

### 2.1 Gas Motion

The flow measurement process is initiated by a gradual closing of the diverter valve to start the upward motion of the bell from its balanced position. A calculation of the net mass flow into the bell during the stroke of the diverter valve is based on the difference between the initial steady state mass flow out of the valve and the smaller flow out during its stroke. If the ports are rectangular and if the stroke is assumed to be one of constant velocity the orifice port area *A*_v_ then can be calculated as *A*_v_=*A*_vi_ (1–γ). Here γ is used as *t/t*_v_, the time *t* from start of the stroke divided by the total stroke time *t*_v_, and *A*_vi_ is the initial open area. For a constant acceleration stroke, *A*_v_=*A*_vi_ (1−γ^2^). The latter assumption is used in the subsequent calculations of the flow as its accomplishment seemed to present a simpler valve design problem, and possibly because it would provide a “smoother” acceleration of the bell upward from its float position. A valve might be designed for a still smoother operation to close slowly at both the beginning and end of its stroke, with a port open area represented, for example, by a function such as *A*_v_=*A*_vi_ (1/2−(1/4) cos^3^πγ+(3/4) cos πγ). The rate of area change with a valve so designed would be ≃0 at the beginning and end. Effects of valve design on prover performance probably will be researched better by calculations than by experiment.

### 2.2 The Ordinary Prover

The net mass flow rate into the bell can be written, using the well known equation for the orifice with coefficient of discharge C_D_, as

$$\begin{array}{c} dm/dt=C_{D}A_{\text{vi}}{\left(2\rho_{i}\Delta P_{i}\right)}^{1/2}\\ -C_{D}A_{\text{vi}}{\left(2\rho_{i}\Delta P\right)}^{1/2}\left(1-\gamma^{2}\right) \end{array}$$ (1)

where Δ*P* is (*P−P*_a_) and Δ*P*_i_ is (*P*i−*P*_a_) and the first term on the right side of eq (1) is the input gas flowrate *FRI.* This rate is to be compared to that established by the calculations from the derived motion of the bell and sealing liquid and for the change of state of the gas in the bell. The mass of gas in the bell and inlet pipe is represented by *m* and *ρ* is its density.

The gas law is now used to relate *P* to the bell and oil motions so that it can be inserted into eq (1). The gas law can be written, using *P*_i_ as the initial balance pressure, as

$$\begin{array}{l} \left(P/P_{i}\right)={\left(mV_{t}/m_{t}V\right)}^{k}\\ ={\left(m/m_{t}\right)}^{k}{\left(V_{t}/\left(V_{t}+Ax+\Delta V\right)\right)}^{k} \end{array}$$ (2)

where *m*_t_ and *V*_t_ are the initial mass and gas volume under the bell and inlet pipe, and *Ax* is the volume change under the bell due to its motion *x* from *x*=0. Δ*V* is the change of gas volume from oil entrainment on the bell as it moves upward plus the volume change caused by motion of the liquid seal. The exponent *k* may be taken as the ratio of gas specific heats when an apparatus is used that would give adiabatic conditions. For a very small apparatus in which the process might be one of almost constant temperature, *k* should approach unity. Note here that *A* is the effective area of a bell prover and is given in [1] as

$$A=A_{1}+A_{2}A_{3}/\left(A_{3}+A_{4}\right)$$ (3)

where *A*_1_ is the interior cross section area of the bell, *A*_2_ the cross section area of the metal of the bell, *A*_3_ and *A*_4_ are the inside and outside horizontal surface areas of the sealing liquid, respectively.

Equation (2) is based first on an assumed uniformity of conditions throughout both volumes *V*_i_ and *V*_b_ which allows use of the heat-exchanger but no metallic-wool or porous-plug. Second, the frequency of a Helmholtz resonator built like the bell and inlet pipe was calculated to range from about 8 to 3 times the expected frequency of the pressure pulsations as expected from the motions of the bell. Resonance at the Helmholtz frequency probably will not be observed experimentally.

The gas volume correction for the entrained oil can be derived from eq (9) in reference [1]^1^ assuming constant pressure (and density) during motion of the bell. Second, if the bell is held stationary while the pressure is changed, the oil seal will move and this also will change the volume of gas under the bell. It is not assumed here that the oil level change will necessarily be such as to maintain equilibrium with the pressure change. Thus Δ*V* in eq (2) can be written

$$\Delta V=\left[\left(A_{3}-A_{4}\right)/\left(A_{3}+A_{4}\right)\right]\sum_{n}\Delta f-A_{3}\left(h_{3}-h_{3,i}\right)$$ (4)

where the summation indicates the progressive effect starting from the initial balance condition for the bell. The entrained oil volume ∑Δ*f* is the volume on one side of the bell, and (*h*_3_−*h*_3i_) represents the change of sealing liquid level from the initial balance position.

Equation (19a) of [1] gives ∑Δ*f* (assumed equal to ∑Δ*f*_3_ and equal to ∑Δ*f*_4_) as

$$\sum \Delta f={\left(4\pi A_{1}\right)}^{1/2}\left(Z_{3}\right)\nu_{0}^{0.644}\sum_{n}{\dot{x}}^{1.644}\Delta t$$ (5)

where *Z*_3_ is a dimensional constant and *n* is a counter for the number of time intervals in the proposed summation process to replace the integral of eq (19a). Here x is the velocity of the bell, and ν_0_ is the kinematic viscosity of the sealing oil. $\dot{x}$ has been used in the summation process assuming it adequately represents the quantity $\left(\dot{x}-{\dot{h}}_{3}\right)$ in eq (19a).

The gas law is used to express the gas density *ρ* in terms of its pressure in the bell and inlet pipe and then the now equation (1) can be written

$$\begin{array}{c} \frac{dm}{dt}=FRI-C_{D}A_{\text{vi}}[2\rho_{i}P_{i}{\left(P/P_{i}\right)}^{1/k}(P/P_{i}\\ {-P_{a}/P_{i})]}^{1/2}\left(1-\gamma^{2}\right). \end{array}$$ (6)

It should be noted that *C*_D_ will be specified as equal to zero at the instant that *γ*= 1 to simulate a closed diverter valve. Equations (4) and (5) can now be used to insert Δ*V* into eq (2) for *P/P*_i_ which will be used in eq (6) above. These three equations contain numerous terms to be derived subsequently in the discussions of the liquid motions and the equations of motion for the bell.

### 2.3 The Modified Prover

A computer simulation of the modified prover’s performance requires that eq (6) be replaced by two now rate equations. The first is an equation for the net rate of accumulation of mass in *V*_b_ and is expressed as

$$dm_{b}/dt=FRI-FRI\left(P_{b}-P\right)\left(P_{\text{bi}}-P_{i}\right)$$ (6a)

where the second right-hand term represents both the mass rate of now from the porous plug and also the mass rate entering the space under the bell. Second, the net rate of accumulation of mass *m* under the bell is

$$\begin{array}{c} dm/dt=FRI\left(P_{b}-P\right)/\left(P_{\text{bi}}-P_{i}\right)\\ -C_{D}A_{\text{vi}}{\left(2\rho \Delta P\right)}^{1/2}\left(1-\gamma^{2}\right) \end{array}$$ (6b)

and again *C*_D_ should be made equal to zero at the instant *γ* is specified to equal unity. Note that (*P*_bi_‒*P*_i_) is the initial pressure drop that is to be specified across the porous plug and that the flow rate through it is proportional to (*P*_b_−*P*). This requires a plug designed for viscous flow which probably would be more effective than one designed for turbulent flow.

An eq (2a) can now be written similar to eq (2) to derive *P* as expressed by the equation

$$P/P_{i}={\left(m/m_{i}\right)}^{k}(V_{i}/{\left(V_{i}+Ax+\Delta V\right)}^{k}$$ (2a)

with the reminder that *P, m, m*_i_ are for conditions under the bell only. For the specified constant gas temperature in *V*_b_, *P*_b_ can be written as

$$P_{b}=P_{\text{bi}}m_{b}/m_{\text{bi}}.$$ (2b)

Equations (4) and (5) are used again in the equations above as in those equations for the ordinary prover.

### 2.4 Liquid Motions and Forces

It is intended here to derive the forces on the bell that are associated with the motion of the bell in the sealing liquid. For this purpose some aspects of Couette flow in both steady and unsteady state motion between parallel plates will be discussed. It is also necessary to look at the development of the laminar boundary layer on a flat plate in the event that this could be dominant as to the force it applies to the bell. These considerations might be useful in examining further the forces from oscillatory liquid motion, but these will be ignored as they tend to cancel when both sides of the bell are considered. However, the liquid oscillation would affect the volume of gas under the bell and an attempt will be made to account for this effect. Other force aspects of the liquid motion, such as from entrained oil on the sides of the bell and motion of the oil at its bottom edge are estimated to include them in the bell’s motion equation.

#### 2.4.1 Couette Flow I

The well-known momentum equation of NS may be used to derive the velocity profile for a fully-developed, steady flow between parallel plates. This condition is practically satisfied if, in figures 1a and 1b, *r* > >2*b* and (*L−x*)> >2*b*. These length conditions would limit the influence of the complex flows that exist at the free surface and at the bottom edge of the bell. Thus the applicable equation is written as

$$\begin{array}{l} -\partial P/\rho_{0}\partial x+\nu_{0}\left(\partial^{2}u/\partial x^{2}+\partial^{2}u/\partial y^{2}+\partial^{2}u/\partial z^{2}\right)\\ =u\;\partial u/\partial x+\nu \partial u/\partial y+\partial u/\partial t \end{array}$$ (7)

where *y* is the distance from the bell’s surface, *z* is along its circumference and *u* and *υ* are the liquid velocities in the *x*- and *y*-directions, respectively, as illustrated in figure 1b. For the specified conditions, the terms ∂*u*^2^/∂*x*^2^, ∂^2^*u*/∂*z*^2^, ∂*u*/∂*x*, υ and ∂*u*/∂*t* in eq (7) are all equal to zero. Two boundary conditions, namely that *u = U* at *y* =0 (or that the liquid on the bell’s surface is carried up with the bell whose velocity is *U*) and second that *u* =0 at *y*=2*b* are now used in the integration of eq (7) which gives the profile as follows:

$$\begin{array}{l} u/U=1-\left(1/2bU\right)\left(U+\left[2b^{2}/\mu_{0}\right]\partial P/\partial x\right)y\\ +\left(1/2\mu_{0}U\right)\left(\partial P/\partial x\right)y^{2}. \end{array}$$ (8)

It should be noted that ∂*P*/∂*x* was assumed to be constant across *y* in the derivation of eq (8).

Equation (8) may now be used to specify a value of ∂*P*/∂*x* such that the liquid up-flow adjacent to the bell is balanced by a down-flow adjacent to the tank wall. Thus, an integration of the flowrate across *y* derives the volume rate total which, when set to zero, gives ∂*P*/∂*x* as

$$\partial P/\partial x=3\mu U/2b^{2}.$$ (9)

The pressure drop along the up-flow as expressed in terms of a liquid head Δ*h* is derived from eq (9) to be equal to 3*µU*(*L*−*x*)/2*b*^2^*ρ*_0_*g.* The up-flow must spill over at the free surface to build a liquid head at the tank wall comparable to Δ*h* to provide for the flow down along the tank wall and for its recirculation at the bottom of the bell. When the value of *∂P/∂x* is inserted into eq (8) the velocity profile for zero net flow is found to be

$$u/U=1+\left(3/4\right)\eta^{2}-2\eta ,\;\eta =y/b\;\text{and}\;Q=0.$$ (10)

Some of these concepts concerning the motion of the sealing liquid are illustrated in figure 1b.

Several interesting features of the flow can now be derived from the velocity profile. First, the velocity gradient at the bell’s surface is

∂u/∂y=−2U/b,y=0andQ=0 (11)

for calculation of the shear stress there. Second, the volume rate of flow (spillover) carried up by movement of the bell, between the surface and the *u* =0 point (*y*=2*b*/3), is calculated to be equal to

Q=0.296ℓbU,y=0toy=2b/3 (12)

for one side of the bell. The quantity *ℓ* is an average circumference of the bell. Third, this volume rate can be compared to the entrainment rate d*f*/d*t* of liquid that adheres to the bell as its surface leaves the liquid. Reference [1] gives this rate for a constant bell velocity, $\dot{x}$, as

$$df/dt≃Z_{3}\ell \nu_{0}^{0.644}{\dot{x}}^{1.644}.$$ (13)

For this prover, the entrainment rate ranges from 1.4 to 6 percent of *Q* in the range of bell velocity researched and discussed in section 3.

The equations of motion will be integrated numerically using a stepwise procedure between time intervals Δ*t.* In a given interval the bell will have velocity 1e8b $\dot{x}\equiv U$ which will be used to calculate force *F*_1_ on the bell during the interval. Equation (11) gives the velocity gradient and when it is multiplied by *µ* and the wall areas involved gives *F*_1_ on the two sides of the bell as

$$F_{1}=-4\;\mu \ell \left(L-x\right)U/b.$$ (14)

#### 2.4.2 Couette Flow II (a)

H. Schlichting [3] in his figure 5.6 and eq 5.24 presents the velocity profile in the fluid between a suddenly accelerated wall and one that is stationary. This “sudden” concept is regarded as applicable here to determine the force on the bell for the integration of the equation of its motion, because the bell velocity changes from interval to interval. His analysis started with two terms of the NS equation, namely the local acceleration term interacting with the friction force. This applies because the convective acceleration and pressure gradient terms are zero in this non-steady, parallel-flow situation.

With that modified NS equation and using appropriate boundary conditions the derived velocity profile is given as a series of complementary error functions (erfc) of the quantities α_1_ [=2*b*/2(*v*_0_*t*)^1/2^] and α [=*y*/2(*v*_0_*t*)^1/2^]. Partial differentiation (with respect to *y*) gives the velocity gradient at the moving wall (*y*=0) as

$$\begin{array}{c} \partial u/\partial y=-\left(\Delta \dot{x}/{\left(\pi \nu_{0}t\right)}^{1/2}\right)(1+2e^{-\left(2\alpha_{1}\right)2}\\ +2e^{-\left(4\alpha_{1}\right)2}\ldots ),y=0 \end{array}$$ (15)

where $\Delta \dot{x}$ represents the velocity “jump” caused by the “sudden” acceleration. The gradient→∞ as *t*→0, and as *t*→∞, the gradient decays to that for the steady moving wall.

It is proposed here to calculate a time-averaged value of the velocity gradient from *t* =0 to *t*=Δ*t*, and to use the “averaged” gradient to calculate force *F*_2_ on the bell. The time-averaged value calculates to be

$$\begin{array}{l} \left(\frac{\bar{\partial u}}{\partial y}\right)=2\Delta \dot{x}/{\left(\pi \nu_{0}\Delta t\right)}^{1/2}-4\Delta \dot{x}e^{-4b^{2}/\nu_{0}\Delta t}/{\left(\pi v_{0}\Delta t\right)}^{1/2}\ldots \\ t=0\;\text{to}\;t=\Delta t \end{array}$$ (16)

where the second and other terms on the right side of eq (16) are negligible for the small values of Δ*t* to be used here. This result is taken as most applicable during the acceleration phase of the motion of the bell. Thus eq (16) leads to

$$F_{2}=-4\mu \ell \;X_{\text{WH}}\Delta \dot{x}/{\left(\pi \nu_{0}\Delta t\right)}^{1/2}$$ (17)

(b) The “sudden” concept is also regarded as applicable to estimate the motion of the oil seal (as caused by pressure changes in the bell) and the resultant change of *h*_3_ as used in eq (4) for the volume of gas under the bell. Here it is the oil that is moved suddenly, not the bell. If the velocity of the oil is constant over most of the space between the bell and the walls of the tank, a simple motion equation can be used to estimate *h*_3_ and *h*_3_ by considering the oil as a solid in motion.

A velocity profile in this “sudden” case is adequately represented (by a single term of a series again for the small time intervals to be used here) as

$$u/{\dot{h}}_{3}≃\left(2/{\left(\pi \right)}^{1/2}\right)\int_{\alpha }^{2\alpha_{1}-\alpha }e^{-\eta 2}d\eta$$ (18)

where the approximate equality sign also signifies that ${\dot{h}}_{3}$ is not exactly constant across the distance 2*b.* The integration limits for *η* are calculated from *α*_1_ and *α* as given previously. A numerical integration of eq (18) for values of *t* assumed equal to integration time intervals Δ*t* to be used here does show an almost constant velocity across practically all of the distance 2*b.* Thus, a value of *h*_3_ derived from the “solid” equation of motion should be reduced by a factor derived from an average value of $u/{\dot{h}}_{3}$ across the distance 2*b*. It should be noted here that the oil motion effect on the gas volume is expected to have most significance while the diverter valve is closing. If the subsequent motion of the bell is at constant velocity the motion of the oil seal should have insignificant influence. Note the big “if” in the previous sentence.

#### 2.4.3 Laminar Boundary Layer

A. H. Shapiro [4] presents information on the development of this layer and its velocity profile which was used to estimate its thickness and possible drag force on the bell. It can be shown that the boundary layer thickness quickly becomes comparable to the separation between bell and tank. This would require large and unrealistic pressure gradients in the region such as to void the computations. These and other considerations lead to the conclusion that the laminar layer cannot build up in the apparatus discussed here.

#### 2.4.4 Other Forces (a)

Sealing liquid that is entrained on the walls of the bell previously was stated to modify the indicated volume of gas collected. Here the liquid acts to modify the mass of the bell by way of the force of gravity on the liquid. This will be the third non-conservative force *F*_3_ calculated below:

$$\begin{array}{l} F_{3}=-2f_{3}\rho_{0}g\\ =-2\rho_{0}g{\left(4\pi A_{1}\right)}^{1/2}\left(Z_{3}\right)\nu_{0}^{0.644}\sum_{n}{\dot{x}}^{1.644}\Delta t. \end{array}$$ (19)

**(b)** Liquid at the bottom edge of the bell is sucked upward by it which creates a drag force there which can be stated as

$$F_{4}=-A_{2}\rho_{0}{\dot{x}}^{2}/2.$$ (20)

### 2.5 Bell and Liquid Motions

The equation of motion (13) of [1] together with the initial conditions and static balance eqs (14), (15), and (16) of [1] will be used here to set up the second order motion equation for the bell. As indicated in section 2.4.2, it appears that the oil motion can be regarded as the motion of a solid and this basis will be used to set up a second-order differential equation of motion for the oil. These equations, in conjunction with the first-order differential eq (6) for the gas motion, will comprise the system of equations to be handled by numerical integration. For this purpose they will be transformed by the usual procedure into five first-order equations.

For the motion of the bell eq (14) of [1] was used first, here with *x*_i_=0, to calculate *h*_4i_ at the initial balance condition using an assumed (or specified) value of (*P*_i_−*P*_a_). Second, eqs (15) and (16) of [1] were used to calculate *M*_3_*/L*_3_ (the chain mass per unit of length) so that the cam and buoyancy compensator *M*_4_ could be replaced with the chain compensator. By this means the term involving ${\dot{x}}^{2}$ is removed from the bell’s equation of motion. The equation thus will be a little simpler. Performance with the chain can be compared later with that of the usual suspension system. Third, eq (13) of [1] then can be simplified further to read as below (after noting that *A*_1_ (*P*_i_−*P*_a_)=*g* (*M*_1_−*M*_2_−*ρ*_0_*A*_2_*L*) −*ρ*_0_*gA*_2_*h*_4i_):

$$\begin{array}{l} \left(M_{1}+M_{2}+I/r_{3}^{2}\right)\ddot{x}=-\left(\rho_{0}A_{2}-2M_{3}/L_{3}\right)gx\\ +A_{1}\left(P-P_{i}\right)+\rho_{0}gA_{2}\left(h_{4}-h_{4i}\right)+\Sigma F. \end{array}$$ (21)

All terms above are zero at the initial balance conditions of *x*=Σ*F*=0 and *h*_4_=*h*_4,i_ and *P=P*_i_ Σ*F* designates the sum of the drag forces. Figure 1 locates and defines some of the quantities used. *I* is the moment of inertia of the pulley.

Two different approaches might be selected to set up the oil motion equation, one regarding the oils on the two sides of the bell as two separate “solids”, or by treating the oil as a single “solid” using the assumption that *A*_3_=*A*_4_. For the two “solids” case the equation is quite complicated and contains a product of the oil acceleration and elevation and also its velocity squared. This seems an unwarranted complication at the present time and the single “solid” will be used.

The equation of motion for the single “solid” is derived using the familiar concept that acceleration is given by force per unit mass and that (*h*_4_−*h*_4i_)≃−(h_3_−h_3i_), to give

$$\begin{array}{c} {\ddot{h}}_{4}=\left(P-P_{i}\right)/\left[2\rho_{0}\left(L-x\right)\right]-g\left(h_{4}-h_{4i}\right)/\left(L-x\right)\\ -\;\text{Force}\;\text{Loss}/\left[\rho_{0}\left(A_{3}+A_{4}\right)\left(L-x\right)\right]. \end{array}$$ (22)

It should be noted here that the oil mass to be moved is *ρ*_0_(*A*_3_+*A*_4_)(*L−x*), and that the term containing *h*_4_ is the liquid’s back pressure. The factor 2 (and also unity in the liquid head term) arises from the pressures acting on the assumed area=(1/2)(*A*_3_−*A*_4_). A “Force Loss” is included in the equation to include the effect of the turn that the oil must make at the bottom of the bell of the ordinary prover. This loss is assumed to be equal to one-half of the velocity pressure, or (1/2)(1/2) $\rho_{0}{\dot{h}}_{4}^{2}$ multiplied by the area *A*_3_, which then gives the last term on the right side of eq (22) above as $\left(1/8\right){\dot{h}}_{4}\left|{\dot{h}}_{4}\right|/\left(L-x\right)$. The quantity $\left|{\dot{h}}_{4}\right|$ is used to make the force loss applicable to both flow directions. When the two orifices are installed to constrict the oil flow space down to an area of (1/2)*A*_5_ on each side of the bell to modify this part of the prover, a pressure drop will be introduced into the flow there. This drop will arise from the frictional loss of mechanical energy mostly on the downstream side of each constriction. For an area constriction down to about 10 percent, a possibly conservative estimate of the pressure drop would correspond to one velocity head pressure based on the liquid’s average velocity in the constricted area. On this bases the last term of eq (22) will be equal to the quantity $\left(1/2\right){\left(A_{3}+A_{4}\right)}^{2}{\dot{h}}_{4}\left|{\dot{h}}_{4}\right|/A_{5}^{2}\left(L-x\right)$ when the orifices are used.

In the context of the discussion above it should be noted that the “solid” oil motion will have some effect on the ordinary bell by the necessity of making the U-turn there. If it’s assumed that the downward motion of the oil pressurizes the “dead” oil space below the bell and acts to help move the bell, the force *F*_5_ can be estimated as

$$F_{5}=\left(1/4\right)\rho_{0}A_{2}{\dot{h}}_{4}^{2}$$ (23)

where the above is based on one-half of the velocity pressure. The above force is replaced, for the modified bell, by a suction force on the bottom of the bell as caused by the downward motion of the oil through each orifice constriction. This motion is induced by the upward motion of the bell. As discussed above for a proposed area constriction in the orifice down to 10 percent, one velocity head pressure loss (based on the velocity in the constriction) is probably a conservative estimate. On this basis *F*_5_ for the modified prover is

$$F_{5}=-\left(1/2\right)\rho_{0}A_{2}{\left(A_{2}/A_{5}\right)}^{2}{\dot{x}}^{2}.$$ (23a)

These forces *F*_5_ are introduced as reminders that they and other “end effect” forces exist but probably have small effects.

### 2.6 Summary of Motion Equations

The gas flow equation (6) for the ordinary prover is

$$\begin{array}{l} \dot{m}=FRI-C_{D}A_{\text{vi}}{\left(2\rho_{i}P_{i}\right)}^{1/2}{\left(P/P_{i}^{1/k}\right)}^{1/2}(P/P_{i}\\ {-P_{a}/P_{i})}^{1/2}\left(1-\gamma^{2}\right) \end{array}$$ (24)

where *C*_D_ = 0 for the condition *γ*≥1 and where the factor (1−*γ*^2^) assumes the use of a constant acceleration diverter valve. Equation (24) needs auxiliary eq (2) for *P/P*_i_ and eqs (4) and (5) for *V/V*_1_ as follows:

$$P/P_{i}={\left[m/m_{t}/\left(V/V_{t}\right)\right]}^{k};$$ (25)

$$\begin{array}{l} V/V_{t}=1+Ax/V_{t}+\left(Z_{1}/V_{t}\right)\sum_{n}\left({\dot{x}}^{1.644}\Delta t\right)\\ +Z_{2}\left(A_{3}/V_{t}\right)h_{4}; \end{array}$$ (26)

where

*Z*_1_ = [(*A*_3_−*A*_4_)/*A*_3_+*A*_4_] (*Z*_3_) (4π*A*_1_)^1/2^ ν_0_^0.644^;
*Z*_2_ = a correction factor for lack of oil “solidity”;
*Z*_3_ = a constant whose value depends on the system of units that will be used.

Note above that *h*_4_ has replaced (*h*_3i_−*h*_3_) because *A*_3_ is assumed equal, in eq (26), to *A*_4_ and that the motion equations when integrated will give *h*_4_=(*h*_4_−*h*_4i_).

The gas flow eqs (6a) and (6b) for the modified prover are:

$${\dot{m}}_{b}=FRI-FRI\left(P_{b}/P_{i}-P/P_{i}\right)/\left(P_{\text{bi}}/P_{i}-1\right);$$ (24a)

and

$$\begin{array}{l} \dot{m}=FRI\left(P_{b}/P_{i}-P/P_{i}\right)/\left(P_{\text{bi}}/P_{i}-1\right)\\ -C_{D}A_{\text{vi}}{\left[2\rho_{i}P_{i}{\left(P/P_{i}\right)}^{1/k}(P/P_{i}-P_{a}/P_{i}\right]}^{1/2}\left(1-\gamma^{2}\right). \end{array}$$ (24b)

Equations (24a) and (24b) need auxiliary eqs (2a) and (2b) for *P/P*_i_ and *P*_b_*/P*_i_, respectively, and eqs (4) and (5) also will be used again in the following equations:

$$P_{b}/P_{i}=\left(P_{\text{bi}}/P_{i}\right)m_{b}/m_{\text{bi}}$$ (25a)

$$P/P_{i}={\left[m/m_{i}/\left(V/V_{i}\right)\right]}^{k}$$ (25b)

$$\begin{array}{l} V/V_{i}=1+Ax/V_{i}+\left(Z_{1}/V_{i}\right)\sum_{n}\left({\dot{x}}^{1.644}\Delta t\right)\\ +Z_{2}\left(A_{3}/V_{i}\right)h_{4} \end{array}$$ (26a)

The equation for the motion of the bell eq (21) is

$$\begin{array}{l} \left(M_{1}+M_{2}+I/r_{3}^{2}\right)\ddot{x}=A_{1}\left(P-P_{i}\right)\\ -\left(\rho_{0}A_{2}-2M_{3}/L_{3}\right)gx+\rho_{0}gA_{2}h_{4}+\sum_{1}^{5}F \end{array}$$ (27)

which uses individual drag forces, as follows:

$$F_{1}=-\left(4\mu \ell /b\right)\left(L-x\right)\dot{x};$$ (27a)

$$F_{2}=-\left(4\mu \ell /{\left(\pi \nu_{0}\right)}^{1/2}\right)\left(L-x\right)\Delta \dot{x}/{\left(\Delta t\right)}^{1/2};$$ (27b)

$$F_{3}=-2\rho_{0}g{\left(4\pi A_{1}\right)}^{1/2}\left(Z_{3}\right)\nu_{0}^{.644}\sum_{n}\left({\dot{x}}^{1.644}\Delta t\right);$$ (27c)

$$F_{4}=-\left(1/2\right)\rho_{0}A_{2}\;{\dot{x}}^{2};$$ (27d)

$$F_{5}=-\left(1/2\right)\rho_{0}A_{2}\;{\dot{h}}_{4}^{2}\;\left(\text{ordinary}\;\text{prover}\right)$$ (27e)

$$F_{5}=-\left(1/2\right)\rho_{0}A_{2}{\left(A_{2}/A_{5}\right)}^{2}{\dot{x}}^{2}\;\left(\text{modified}\;\text{prover}\right).$$ (27f)

The equation of motion eq (22) for the sealing oil in the ordinary prover is

$$\begin{array}{l} {\ddot{h}}_{4}=\left(P-P_{i}\right)/\left[2\rho_{0}\left(L-x\right)\right]-gh_{4}/\left(L-x\right)\\ -\left(1/8\right){\dot{h}}_{4}|{\dot{h}}_{4}|/(L-x) \end{array}$$ (28)

and for the modified prover the last term on the right side of eq (28) is specified as equal to $50{\dot{h}}_{4}|{\dot{h}}_{4}\left(L-x\right)$ when *A*_5_ is constructed to be equal to 0.1 (*A*_3_*+A*_4_). With this substitute, last term of eq (28) in place we may label the motion equation for the sealing oil as eq (28a).

Equations (24), (27), and (28) or their (a) and (b) substitutes for the modified prover, comprise the two systems of equations to be integrated numerically by the fourth-order Runge-Kutta method as outlined in [2].

A recapitulation of the main assumptions used in the derivations of the above equations seems advisable. It does not seem advisable at the present time to develop a change to eq (24) to account for a possible non-constant value of *C*_D_. Equations (27b) and (28), and the far right-hand term of eq (26) are based on a special integral of the NS equation for the case of “sudden” motion of the bell and of the sealing liquid. Equation (27a) was derived from the NS eq (7) for steady flow between parallel plates without consideration of end effects. Equation (27e) and the far right-hand term of eq (28) but not for eq (28a) are used as reminders of end effects not fully accounted for. The forces of these “estimated” end effects turned out to have an insignificant effect on the calculated performance of the prover. Except in eqs (27), (26a) and (3), *A*_3_ was assumed equal to *A*_4_ with corresponding assumptions *f*_3_=*f*_4_ as in eq (27c) and −*h*_3_=*h*_4_ as in eq (28). And, finally, the flow rate into the bell of the ordinary prover is assumed to be constant and unaffected by the probable small changes of state of the gas in *V*_b_.

## 3. Calculated Results

**Figure 2:**
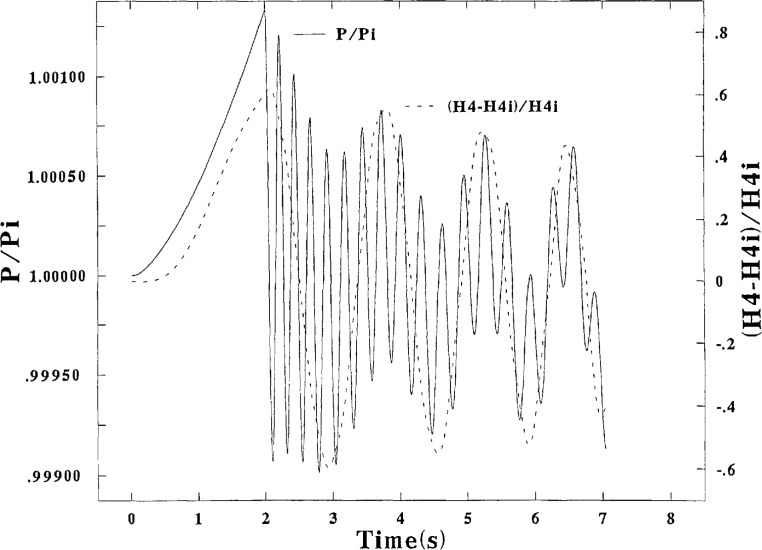
Bell pressure, and height of sealing liquid, plotted vs time. Program HIFLO.

**Figure 3:**
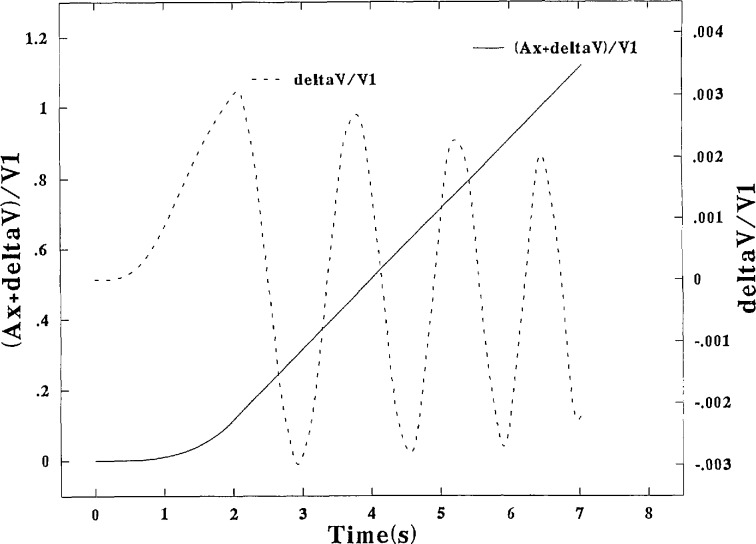
Actual volume of gas collected, and volume correction, plotted vs time. Program HIFLO.

**Figure 4:**
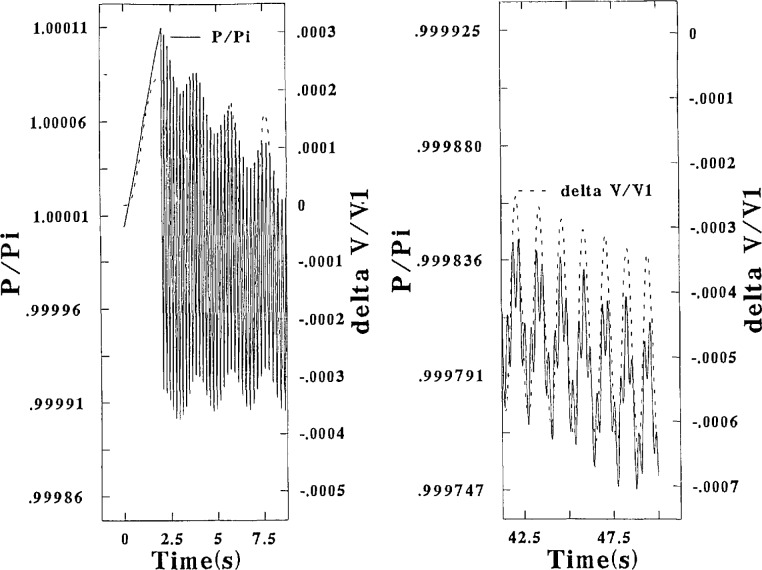
Bell pressure, and gas volume correction, plotted vs time. Program LOFLO.

**Figure 5:**
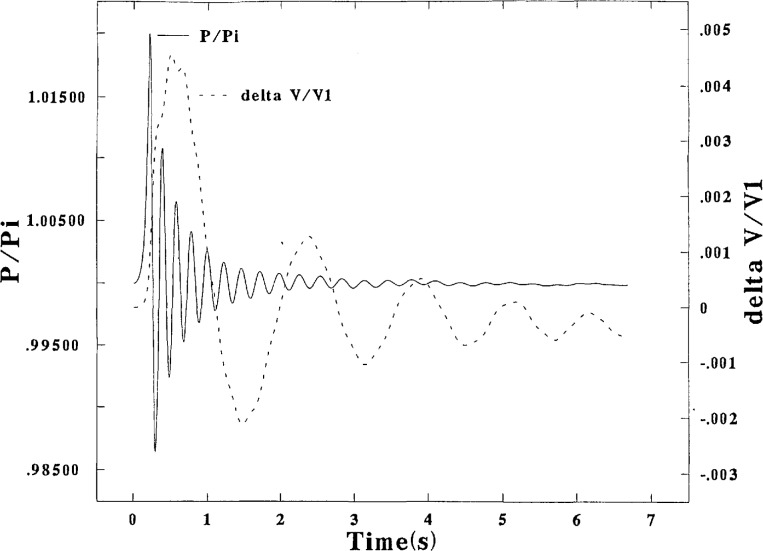
Bell pressure, and gas volume correction, plotted vs time. Program HOLHOG.

The numerical integrations of the equations of motion are used to derive the performance of the provers which will be illustrated by various curves showing the motions of the bell and sealing oil and the derived gas pressure and gas volume correction all expressed as a function of the time. (See figs 2 to 5). These figures can give a good qualitative illustration of the performance, but further computations are necessary to derive quantitative evaluations and comparisons of the measurement performance. Equation (2) can be rearranged for this purpose so that the true mass collected can be expressed in terms of *ρ*_i_*A* (*x*_2_−*x*_1_) with corrections applied to it for the fluctuations of *P* and Δ*V.* Subscripts 1 and 2 are used on *x* to signify values at the beginning and end of the chosen timing interval, respectively. It seemed preferable to separate the *P* and Δ*V* corrections, and to do this the pressure ratio in eq (2) was expressed as very closely equal to (1+(*P−P*_i_)*kP*). With some use of algebra, eq (2) can be written for the modified prover in the form

$$\begin{array}{l} \left(m_{2}-m_{1}\right)≃\rho_{i}A\left(x_{2}-x_{1}\right)[1+\frac{\Delta V_{2}-\Delta V_{1}}{A\left(x_{2}-x_{1}\right)}\\ +\frac{\left(P_{2}-P_{i}\right)\left(V_{i}+Ax_{2}\right)}{kP_{i}A\left(x_{2}-x_{1}\right)}-\frac{\left(P_{1}-P_{i}\right)\left(V_{i}+Ax_{1}\right)}{kP_{i}A\left(x_{2}-x_{1}\right)}\\ +\frac{\left(P_{b2}-P_{b1}\right)V_{b}}{\left(k=1\right)P_{\text{bi}}A\left(x_{2}-x\right)}]. \end{array}$$ (29)

and when the ordinary prover is used *V*_b_ = 0 in the far right-hand term and *V*_i_ should be replaced by *V*_t_ which is given by (*V*_i_+*V*_b_).

### 3.1 The Ordinary Prover

Almost complete physical data were available to the authors on a bell prover of nominal capacity *V*_1_ of 5 ft^3^ (0.1415851 m^3^) and these were used in three computer runs for the first look at the utility of the proposed analytical method. Results of these runs indicate the effectiveness of the integration method as well as demonstrate the size and sources of errors likely to be encountered with the usual laboratory procedures. The prover specifications are listed in table 1 together with data on initial balance conditions, fluid properties and the computer run conditions used in the programs HIFLO and LOFLO. Nominal measured stroke times are 5 and 50 s, respectively, for these two programs.

The two runs at the high rate of flow (nominal collection time of 5 s) were done with different time intervals in the integration process, namely 0.002 and 0.01 s. Rates of flow derived from use of these two integration processes differed by about 0.01 percent. Comparison of other physical variables also showed differences near 0.01 percent. On the bases of this good agreement it was decided to use the 0.01-s time interval for the low (50 s collection) flow rate calculation. It seemed reasonable to expect much larger differences than 0.01 percent between prover performance at the two rates of flow.

Figures (2) and (3) are graphical representations of the calculated results at the high flow rate and figure (4) presents results at the low flow rate. Figure (2) shows a relatively large initial fluctuation of the pressure, about ±46 percent of Δ*P*_i_ from *P*_i_ with a relatively long period, initially about 1.8 s and decreasing to about 1.1s. The pressure fluctuation decreases to about ±30 percent of Δ*P*_i_ from the mean pressure, but the curve near 7 s shows a hint of a possible increase at that point. Higher frequency fluctuations of the pressure, with periods of about 0.3 s, are also evident. The amplitude of the oil motion as converted from values of *h*_4_ to pressure is about 120 percent of the pressure fluctuation. It is evident that the pressure fluctuations are strongly modulated by the oil fluctuations. It is also strongly evident from figures 2 and 3 that the gas volume correction Δ*V* under the bell fluctuates practically in unison with *h*_4_. This fact suggests that failure to measure *h*_4_ possibly can be the major source of error.

At the low rate of flow figure 4 shows a much smaller initial fluctuation of pressure, about ±4.0 percent of Δ*P*_i_ from *P*_i_, and again a relatively long period initially of about 1.8 s which decreases to about 1.4 s. The pressure fluctuation and the pressure level both decrease, the former to ±1.3 percent of Δ*P*_i_ about the mean pressure which is about 9 percent of Δ*P*_i_ below *P*_i_. These fluctuation amplitudes are about six percent of those at the high rate of flow, as compared to the flowrate ratio of 10 percent. The amplitude of the sealing liquid motion (not shown) begins at about 115 percent of the pressure, but winds up near the end of the measurement stroke at about twice that of the pressure fluctuation. As at the higher flow rate, the gas volume correction (not ordinarily measured) fluctuates practically in unison with *h*_4_ and with the low frequency pressure fluctuations. The high-frequency fluctuations have periods of about 0.19 s which increases to about 0.31 s.

The high frequency motion calculated for the bell can be shown to be real by considering the bell to be vibrating as if suspended from a “spring” which can “replace” the air enclosed in the bell. For the case of the very low values of the pressure fluctuations prevailing here, this concept is sufficiently accurate to derive the “spring constant” on *x* as equal to $kA_{1}^{2}P_{i}/V$ where *V* is the volume of air and *x* is the motion of the bell about a stationary position. The vibration period for this well known case is $2\pi /{\left\{kA_{1}^{2}P_{i}/\left[V\left(M_{1}+M_{2}+I/r_{3}^{2}\right)\right]\right\}}^{1/2}$ which calculates to be in the range from 0.2 to 0.4 s. Inclusion of the buoyancy force and damping (drag) forces would not change the calculated period sufficiently to warrant the added complication. Here again these periods are in conformity with those derived from the full motion analysis.

An inspection of eq (29) and figures 2, 3, and 4 demonstrates the problems involved with making accurate measurements of the fluctuating physical variables and also the need to correlate those measurements with the timing points used to define the stroke of the bell. It is also evident that various choices and measurement methods can be used to try to derive the various corrections in eq (29) for the true collected mass.

#### 3.1.1 Method 1

If the measurements of *P* and either Δ*h*_4_ (or Δ*V*) are accurate at any instant and accurately correlated with the bell positions *x*_1_ and *x*_2_, eq (29) provides the true mass.

#### 3.1.2 Method 2

Figure 2 demonstrates that no corrections to *ρ*_i_*A*(*x*_2_−*x*_1_) are necessary if the initial and final timing points (at 3.36 and 6.2 s, for HIFLO) can be identified by the instrumentation when the variables of (*P−P*_i_) and Δ*h*_4_ both equal to zero at both bell positions (a zero-zero criterion). In the more general case (LOFLO, for example) this criterion does not prevail. However, pressure transducers probably are available with capability to follow the low frequency fluctuations of the pressure (as caused by the motion of the oil) with good accuracy especially at the fluctuation midpoints. If the timing points *x*_1_ and *x*_2_ are made to coincide in time with those mid-point pressures, the corrections derived with eq (29) would also be accurate because the mid-points of the Δ*V* Fand the Δ*P* motions are shown here to coincide. These methods should be used but apparently are not because of the practical difficulties. A third procedural compromise, Method 3, is usually used to derive the corrections.

#### 3.1.3 Method 3

Measurements are made with a water manometer to give (what is believed to be) mid-point values of the high-frequency fluctuating pressures (*AFP*) at two preselected, convenient positions *x*_1_ and *x*_2_. These measured *AFP* values are used in eq (29) along with calculated values of Δ*V* (based on *AFP*) to derive “corrections” to *ρ*_i_*A*(*x*_2_−*x*_1_). The “worst” timing point choices that can be derived from figures 2 and 4 are used to illustrate in table 2 the errors that might result with use of Method 3. “True” corrections (of Method 1) are also listed for comparison in Table 2.

For program HIFLO, one “worst” timing point choice would need a very large true correction, while the correction based on values of the *AFP* is only 32 percent of what is actually needed. For a LOFLO “worst” choice, use of the *AFP* gives a correction that is 70 percent of the true correction. The net result is that flow rates based on *AFP* values would be higher than actual by 0.56 and 0.02 percent for the flow rates HIFLO and LOFLO, respectively. It should be noted the corrections for entrained oil on the sides of the bell amounted to −0.013 and −0.003 percent for those two rates. These are not shown above because they are included in the values of Δ*V* in the true correction procedure [see eq (4)]. It should also be noted that other “worst choices” would give corrections of opposite sign.

There are three suggestions to be made concerning possibilities for improving the instrumentation and/or measurement procedures used with the ordinary prover.

#### 3.1.4 Suggestion 1

Instrumentation problems would probably prevent implementation of the ideal situation in which Method 1 could be effectively used. However, experiments should be conducted to try to overcome these difficulties.

#### 3.1.5 Suggestion 2

It might be possible to implement the zero-zero criterion as described in method 2 to use what is essentially a “correctionless” procedure. Two requirements are necessary for this purpose. First, the prover must be modified to maintain the average of the maximum/minimum pressures, *P*_m_, constant and equal to *P*_i_ during the stroke of the bell. Second, an instrument must be available to be able to identify bell positions where the fluctuating values of Δ*P* and Δ*h*_4_ are equal to zero. The first requirement probably can be met by an adjustment of the buoyancy weights (downward by about one and seven percent, respectively, as computed from the pressure curves for HIFLO and LOFLO). This should be and is confirmed if some bell positions, particularly near *x*_2_, can be found where *AFP* values or transducer measured values are equal to *P*_i_. These positions need not necessarily be those that are finally used for the rate measurements.

An instrument which might be used for phase indications of Δ*P* and Δ*h*_4_ is the optical encoder used by G. Kulin, P. Huang, and G. Mattingly [5] to better measure the volumetric displacement of the bell and its velocity. It was mounted on the pulley and it gave (almost) linear vertical motion in the form of electrical pulses. Their temporal traces of the encoder’s output voltage displayed fluctuations of the bell’s velocity at approximately the same period of about 1.8 s as calculated herein. Some computations for program LOFLO (not shown) demonstrated that maximum bell velocity occurred when both Δ*V* and values of (*P*−*P*_i_) both were zero. Therefore, presumably, the encoder’s indication of the maximum bell velocity also occurs at the zero-zero condition. This leads to the suggestion that the timing points should be made to coincide with the maximum velocity indications of the optical encoder to try to eliminate the otherwise needed, but in accurate, *AFP* corrections to derive the true mass of gas collected. As an alternative to the above, more attention should be given to fully implement Method 2.

#### 3.1.6 Suggestion 3

It appears to be possible to implement the zero-zero criterion as described in Method 2 using the *AFP* procedure of Method 3 which can be modified to measure the true collected mass of gas. Two modifications appear of possible utility, both needing a constant value of *P*_m_=*P*_i_ during the stroke of the bell. The first would use computed motions of Δh_4_ (as described herein) to identify and use the bell positions *x*_1_ and *x*_2_ where Δ*h*_4_ values are expected to be zero. *AFP* values would then also equal *P*_i_ and corrections to *ρ*_i_*A*(*x*_2_−*x*_1_) would also equal zero. Results from use of this method could then be compared to results obtained below with the second modified *AFP* procedure.

The second modified *AFP* procedure would utilize a multiplicity of strokes of the bell each with positions *x*_1_ and *x*_2_ systematically moved until both give *AFP* values equal to *P*_i_. *P*_m_ must also be maintained equal to *P*_i_ with this method (see Suggestion 2). This method is based on the assumption that *AFP* values of pressure fluctuate about *P*_i_ with a smaller amplitude than those of the high frequency pressure fluctuations, and with a period of the low frequency fluctuations of Δ*h*_4_. It’s expected that on average five different strokes of the bell would be needed with *x*_1_ and *x*_2_ each moved each time a distance corresponding to the distance traveled by the bell during 0.05 period of the Δ*h*_4_ fluctuation. It’s assumed in the experimental situation that the pressure measuring instrument actually does indicate the *AFP* values. If all of these criteria prevail the modified, multiple *AFP* method is also correction less. An advantage of these two procedure modifications is that neither requires apparatus changes.

#### 3.1.7 The Modified Prover

Figure 5 presents a graphical picture of the calculated temporal traces for the pressure and gas volume correction as derived in program HOLHOG which was specified to have the same flow rate as in HIFLO. These results show that the modifications made to the prover caused large reductions in the amplitude of the pressure and oil motions (particularly for the latter) as was intended. Each oil orifice had a 10 percent open area, the drop across the Porous Plug was specified as equal to 40Δ*P*_i_, and *t*_v_ was reduced to 0.2 s (from 2 s as used in HIFLO and LOFLO). These three changes were made simultaneously and “almost” arbitrarily, and hence the name HOLHOG was chosen. Note that Δ*V* increased by only about 50 percent over that in HIFLO in spite of the 14-fold increase of Δ*P* at the end of 0.2 s. These larger upsurges, however are subsequently efficiently dampened. Although the frequencies of the motions of Δ*V* and Δ*P* are the same as in the ordinary prover, the practically constant phase difference between the two motions is not evident here.

The calculated “worst” corrections for HOLHOG listed in the table above demonstrate that the correction remaining and needed after application of the *AFP* Method 3 is gratifyingly reduced to 0.053 percent from the 0.586 percent needed for the ordinary prover. This possible “worst” result for HOLHOG is improved to a needed remaining correction of only 0.006 percent if a “best” timing choice happens to be made. Phase differences between the Δ*V* and Δ*P* motions that change during the stroke of the bell limit suggestions for improvement in methodology to Suggestion 3 above. However, the need for improvement is much, much less than for HIFLO at its worst.

It’s appropriate at this point to discuss the “almost” arbitrary group of apparatus changes (specified for HOLHOG) and the problem of setting the initial conditions. As a preliminary to HOLHOG, computations of the effect of the orifice installation alone, and next of the effect of the porous plug alone, indicated that dampening was not effective enough before the bell reached *x*_1_ to make a significant improvement. A third preliminary calculation was done to evaluate the effect of a single change in an initial condition, namely a reduction of *t*_v_ from 2 to 0.2 s in program HIFLO. This change produced a two-fold increase in the initial upsurge of Δ*V* over the already large increase evident in HOLHOG. This large increase illustrates the importance of the initial condition, and second that the orifice effectively dampened the motion of the oil in HOLHOG and especially the initial upsurge motion.

Other changes of initial conditions, such as in the flow rate characteristics of the diverter valve, and changes in the relationship of *V*_i_ to *V*_b_ to *V*_1_, also could have significant effects on the performance of both provers studied here. This discussion should now revert back to the need to use a motorized and stroke-designed diverter valve as previously suggested herein. This discussion also leads to the conclusion that prover performance cannot be adequately specified until the initial conditions are too.

## 4. Conclusion

It appears that the equations of motion for the moving elements of the prover system and the procedure used in their numerical integration can produce calculated results which offer a reasonable chance that they can be used to design improvements in the apparatus and measurement procedures. “Dynamic errors” in the measurement of flow rates can arise primarily from the fluctuating motions of the sealing liquid, second from fluctuating velocity of the bell, and third and usually least in magnitude from entrainment of liquid on the bell. Proposed additions to the prover can be used to (1) assure needed equality between gas and bell temperatures and (2) to reduce the amplitude of the fluctuating motions as one way to reduce the magnitude of measurement errors. Three other suggestions for improved measurement procedures include an ideal situation in which pressures and oil motions are accurately measured at any two bell positions of choice. A second more practical suggestion would require that the timing points for the bell motion be properly correlated with indications of the mid-point pressures from a pressure transducer or correlated with indications of the maximums in the fluctuating bell velocity. A third suggestion for improved accuracy entails the utilization of two different approaches both being modifications of that currently in use which relies on manometer reading of the average indications of the high-frequency fluctuating pressures at the two bell positions of choice. Some changes in the initial conditions produced performance changes which emphasizes that blanket performance and accuracy statements cannot be specified unless all initial conditions are also specified.

## 5. Nomenclature

## Figures and Tables

**Table 1:** Prover specifications and computer run conditions

Prover specifications^a^							

*A*	*A*_1_	*A*_2_	*A*_3_		*A*_4_	*ℓ*	

.2238913	.2183231	.01189164	.05481303		.06224530	1.678537 SI	
2.409936	2.35	.128	.59		.67	5.507 U.S.	

*M*^c^	*M*_1_	*M*_2_	*M*_3_*/L*_3_^c^		*M*_4_	$I/r_{3}^{2c}$	

94.71400	99.23840	85.45006	5.569795		0	5.032009 SI	
6.49	6.8	5.855197	.1163280		0	.3443026 U.S.	

*b*	*V*_b_^c^	*A*_5_/(*A*_3_*+A*_4_)	*V*_1_				

0.0175	0.0062	0.1	0.14 m^3^ SI				
0.0575	0.22	0.1	5 ft^3^ U.S.				

Initial conditions and fluid properties							

*L*^c^	*g*	*P*_a_	(*P*_i_−*P*_a_)		*h*_4i_	*x*_i_^c^	

.82296170	9.8054	101362.1	239.4003		1.344412(10^−2^)	0 SI	
2.7	32.17	2117	5		4.410790(10^−2^)	0 U.S.	

*V*_i_^c^	*m*_i_	*k*^c^	*v*_0_		*µ*_0_		

2.208728(10^−2^)	3.409868(10^−2^)	1.4	.2006714(10^−4^)		170.453(10^−4^) SI		
0.78	2.336505(10^−3^)	1.4	2.16(10^−4^)		3.56(10^−4^) U.S.		

*ρ*_0_	*ρ*_a_	*ρ*_i_	(*P*_bi_−*P*_i_)^c^				

850.3685	1.201339	1.204176	40(*P*_i_−*P*_a_) SI				
1.65	2.331(10^−3^)	2.336505(10^−3^)	40(*P*_i_−*P*_a_) U.S.				

Computer run conditions							

Program name	$\dot{m}=FRI$	*t*_v_	*Z*_2_	Δ*t*	$60\dot{m}/\rho_{a}$	Nominal Collection time, $\rho_{a}\;V_{1}/\dot{m}$	

HIFLO^b^ {	3.4018341(10^−2^)	2	.95	.002	1.699021	5 s	SI
	2.331(10^−3^)	2	.95	.002	60 scfm	5 s	U.S.
HOLHOG	Same as above	.2	.95	.0002	––––same as above––––		
LOFLO {	3.4018341(10^−3^)	2	.90	.01	.1699021	50 s	SI
	2.331(10^−4^)	2	.90	.01	6 scfm	50 s	U.S.

a SI units are kilogram, meter, newton and seconds (MKS). U.S. units are slug, foot, pound-force, seconds.

b Program HIFLO was also done with an integration time interval Δ*t* of 0.01 s.

c Derived or assumed as probably applicable.

**Table 2:** Measurement corrections for worst timing choices

Program	Correction Method	Δ(Δ*V*)%	Δ(Δ*P*)%	Δ*P*_b_%	% Total, correction	*t*_1_	*t*_2_
HIFLO	1, True	−0.752	−0.072	NA	−0.824	3.73	6.95
HIFLO	3, AFP	−0.196	−0.065	NA	−0.261	3.73	6.95
HIFLO	Remaining error after AFP Correction = −0.563						
LOFLO	1, True	−0.017	−0.060	NA	−0.077	3.8	44.6
LOFLO	3, AFP	−0.053	−0.001	NA	−0.054	3.8	44.6
LOFLO	Remaining error after AFP Correction = −0.023%						
HOLHOG	1, True	−0.138	−0.045	−0.001	−0.184	3.73	6.6
HOLHOG	3, AFP	−0.108	−0.022	−0.001	−0.131	3.73	6.6
HOLHOG	Remaining error after AFP Correction = −0.053%						

## Footnotes

1 Note that the last term of eq (9) in reference [1] has a typographical error. A ratio of densities, not a product, is correct.

## Abbreviations

*A*: =See eq (3), effective bell area
*A*_1_: =Cross-sectional, open area of the bell
*A*_2_: =Horizontal cross-sectional area of the metal wall of the bell
*A*_3_: =Horizontal area of the inside sealing liquid
*A*_4_: =Horizontal area of the outside sealing liquid
*A*_5_: =Sum of orifice open areas in sealing liquid spaces
*A*_v_: =Area of ports of the diverter valve
*AFP*: =An average or mid-point values of the fluctuations of *P*
2*b*: =Separation distance between bell and tank
*C*_D_: =Coefficient of discharge for the diverter ports
Δ: =Change of
*F*: =Force
*FRI*: =Gas flowrate into the $\text{bell}={\dot{m}}_{i}$
*f*: =Volume of entrained oil on one side of the bell
*g*: =Local acceleration of gravity
*h*_3_,*h*_4_: =Sealing liquid elevations with reference to *L*
Δ*h*: =Oil “spillover head”
*I*: =Moment of inertia of the pulley
*k*: =An average ratio of isothermal to adiabatic compressibility—for air between 1.0 and 1.4
*L*: =Sealing liquid depth when *x*, =0
*L*_3_: =Length of the chain
*ℓ*: =Circumference of bell
*M*: =Mass of the bell
*M*_1_: =Mass of the bell plus that portion of the chain which is on the same side of the pulley when *x*, =0
*M*_2_: =Mass of the counterweight plus its chain weight
*M*_3_: =Mass of the chain
*M*_4_: =Mass for buoyancy compensation (not used here)
*m*: =Mass of the gas in the bell
*P*_m_: =Average of the maximum/minimum pressures in the bell
*P*: =Pressure in the bell
*P*_b_: =Pressure in the bell inlet pipe of the modified prover
Δ*P*: = Differential pressure
*Q*: =Volume rate of spillover liquid on one side of the bell
s: =seconds
*t*: =Time, seconds
*t*_v_: =Diverter valve closing interval, seconds
Δ*t*: =Integration time interval
*t*_1_: =Time at beginning of timing interval
*t*_2_: =Time at end of timing interval
*U*: =Bell velocity along *x*, or “freestream” velocity down along *X*
*u*: =Velocity of sealing liquid to positive *x*
*V*_i_: =Initial volume of gas under the bell only
*V*: =Volume of gas in the bell including *V*_i_ or *V*_t_
*V*_b_: =Volume of gas in the bell inlet pipe
*V*_1_: =Nominal prover capacity
*V*_t_: =*V*_i_+*V*_b_
Δ*V*: =Gas volume correction
υ: =Velocity in the *y*-direction
*x*_1_: =Bell position at beginning of timing interval
*x*_2_: =Bell position at end of timing interval
*x*: =Bell’s movement upward from zero position
*y*: =Distance from bell’s surface
*z*: =Distance around circumference of the bell
*Z*_1_: =A group of constants in eq (26a)
*Z*_2_: =A correction factor for lack of oil “solidity”
*Z*_3_: =1.09 m^−0.93^ s^1.29^ (SI) or 0.36 ft^−.93^ s^1.29^ (U.S.)
*α*: =*y*/[2(*v*_0_*t*)^1/2^]
*α*_1_: =2*b*/[2(*v*_0_*t*)^1/2^]
*γ*: =t/t_v_
*η*: =y/b. Also, value of variable in eq (18)
*µ*: =absolute viscosity of the sealing liquid, force second/length^2^
*v*_0_: =kinematic viscosity of the sealing liquid, length^2^/second
*ρ*: =Liquid or gas density

**Subscript**
a: =Ambient condition or air
b: =conditions in inlet pipe
i: =Initial value, balanced condition
n: =Integration time interval counter
o: =Liquid
v: =Diverter valve

**Superscript**
': =First derivative with respect to time
'': =Second derivative with respect to time

**Dimensions Used**
slug: (=14.593883 kg)
foot: (=0.3048009 m)
lbf: (=4.4482290 N)
lbf/ft^2^: (=47.880056 N/m^2^)
